# Wedelolactone Attenuates N-methyl-N-nitrosourea-Induced Retinal Neurodegeneration through Suppression of the AIM2/CASP11 Pathway

**DOI:** 10.3390/biomedicines10020311

**Published:** 2022-01-28

**Authors:** Kevin Harkin, Josy Augustine, Alan W. Stitt, Heping Xu, Mei Chen

**Affiliations:** Wellcome-Wolfson Institute for Experimental Medicine, School of Medicine, Dentistry & Biomedical Science, Queen’s University Belfast, Belfast BT9 7BL, UK; k.harkin@qub.ac.uk (K.H.); J.Augustine@qub.ac.uk (J.A.); a.stitt@qub.ac.uk (A.W.S.); heping.xu@qub.ac.uk (H.X.)

**Keywords:** neurodegeneration, neuroprotection, inflammasome, Caspase-11, Aim2, IL18, therapeutic effect of Wedelolactone

## Abstract

N-methyl-N-nitrosourea (NMU) is widely used to model oxidative stress and inflammation mediated retinal neurodegeneration. Wedelolactone (WD) is known to have antioxidant, anti-inflammatory, and neuroprotective roles. This study tested the therapeutic potential of WD in NMU-induced retinal neurodegeneration and investigated the underlying mechanisms in mice. NMU (40 mg/kg) was injected intraperitoneally into C57BL/6J mice with/without an intravitreal injection of WD (1 μL/eye, 200 μM). Seven days later, retinal function and structure were evaluated by electroretinography (ERG) and Spectral Domain Optical Coherence Tomography (SD-OCT). The expression of inflammasome components (*Aim2*, *Caspase 1/11*, and *Il1b/Il18*) in the total retina lysate was evaluated by RT-qPCR. In vitro, 661W photoreceptor cells were transfected with synthetic double-strand DNA (Poly(dA:dT)) with/without WD pre-incubation. The aim2-related inflammasome expression was evaluated by RT-qPCR and immunocytochemistry. The production of IL18 was measured by ELISA. NMU treatment significantly impaired A- and B-wave response (ERG) and reduced neuroretina thickness (OCT). This was significantly attenuated upon intravitreal injection of WD. The expression of *Aim2*, *ACasp1*, and *Casp11* was increased in the retina from NMU-treated mice, and this was prevented by WD treatment. Transfection of Poly(dA:dT) upregulated *Aim2, Casp11,* and *Il18* expression in 661W cells. WD prevented their upregulation and reduced IL18 production. Aim2 inflammasome activation is critically involved in NMU-induced retinal neurodegeneration and WD can protect the retina particularly through the suppression of this inflammasome-linked pathway.

## 1. Introduction

The degeneration of retinal photoreceptors is the ultimate cause of vision loss in retinal degenerative diseases such as retinitis pigmentosa (RP) [1], age-related macular degeneration (AMD) [2,3], and retinal detachment (RD). Photoreceptors have high oxygen-dependency, reflected by their abundance of mitochondria [4,5]. Mitochondria sequester towards the inner segment of photoreceptors, where they are in the closest proximity to the oxygen supplied by the choriocapillaris and deep retinal vasculature [6]. While mitochondria are critical to meet photoreceptor ATP requirements, in some disease states, the dysfunction of these organelles can lead to the release of damaging free radicals or when protective mechanism are compromised, they can themselves become subject to oxidative species. Indeed, oxidative stress mediated photoreceptor damage is involved in the pathogenesis of various retinal diseases such as, AMD [7], Glaucoma [8], and DR [9]. Specifically, oxidative stress-induced mitochondrial dysfunction has been found in glaucoma [8] and DR [10], as well as other conditions such as Alzheimer’s disease [11] and sepsis [12]. In such settings, the mitochondria can manifest membrane permeability and instability of the mitochondrial DNA (mtDNA) [13,14] causing leakage of mtDNA into the cytosol. Cytosolic mtDNA may engage with absent in melanoma 2 (AIM2) and the nucleotide oligomerisation domain (NOD), leucine-rich repeats, and pyrin domain-containing protein 3 (NLRP3) inflammasome complexes [15,16] leading to the activation of their signalling cascades. This then induces the cleavage of Caspase-1 or -4 (Caspase-11: murine homolog), which subsequently leads to the maturation and secretion of pro-inflammatory cytokines interleukin (IL)1B and IL18 [17,18,19]. The activation of these pro-inflammatory cytokines has the potential to trigger a highly inflammatory form of cell death known as pyroptosis [20]. Presently, there are no drugs that can successfully treat these retinal degenerative diseases, and approved drugs only relieve symptoms.

There are several rodent models of photoreceptor degeneration, including transgenic models (e.g., Rd gene mutations) [21], light-induced [22], and chemical-induced (e.g., N-methyl-N-nitrosourea (NMU)) photoreceptor degeneration [23]. Apoptosis has been the most widely described mechanism of photoreceptor cell death in these models, as first described by Cook et al. [24], whereby positive terminal deoxynucleotidyl transferase dUTP nick end labelling (TUNEL) staining was seen within the photoreceptors of cats subjected to RD. As oxidative stress and inflammation are believed to play an important role in various models of photoreceptor degeneration, we hypothesised that oxidative damage in photoreceptors may release mtDNA, which may stimulate inflammasome activation through various cytosolic DNA sensors. WD is a natural occurring coumestan found in *Eclipta alba* and has been reported to have anti-oxidative [25,26] and anti-inflammatory [27,28,29] effects in acute liver injury and renal fibrosis. Additionally, WD has shown to have neuroprotective effects in a model of sporadic amyotrophic lateral sclerosis [30]. In this study, we evaluated the therapeutic effect of WD in NMU-induced retinal neurodegeneration. We found that WD strongly protected photoreceptors from NMU-induced neurodegeneration. Mechanistically, WD suppressed Aim2/Casp11 inflammasome activation.

## 2. Materials and Methods

### 2.1. Animal Maintenance

C57BL/6 J (WT) mice were maintained in the Biological Service Unit (BSU) at Queen’s University Belfast and had free access to food and water ad libitum. In vivo procedures and levels of anaesthesia were conducted under the regulation of the UK Home Office Animals (Scientific Procedures) Act 1986 and were approved by the local Animal Welfare and Ethical Review Board (AWERB). All procedures were compliant with the Association for Research in Vision and Ophthalmology (ARVO) Statement for the use of Animals in Ophthalmology and Vision Research.

### 2.2. Induction of Photoreceptor Degeneration and WD Intravitreal Injection

Photoreceptor degeneration was induced by intraperitoneal injection of 40 mg/kg of NMU (Fluorochem, Glossop, UK) into 3-month-old mice. In the WD treatment groups, mice received 1 µL of WD (200 µM) (Cayman Chemical, Ann Arbor, MI, USA) or 1 µL of DMSO (0.1% DMSO in PBS) (Sigma-Aldrich, Gillingham, UK) through intravitreal injection [31] immediately before NMU injection. Briefly, the mice were anaesthetised via isoflurane inhalation and their pupils were dilated with 1% atropine sulphate and 2.5% phenylephrine hydrochloride (Chauvin, Essex, UK). A 33G needle connected with a microsyringe repeating dispenser (Hamilton Bonaduz AG, Bonaduz, Switzerland) was inserted via the pars plana into the vitreous under a surgical microscope (Nikon UK Ltd., Surrey, UK).

### 2.3. Electroretinogram (ERG)

Ganzfeld ERG was performed using an Espion visual electrophysiology system (Diagnosys LLC, Littleton, MA, USA), according to manufacturer’s instructions and as previously described by our group [31,32]. Briefly, four responses were averaged at each light intensity (0.8, 2.5, 8, and 25 cdxs/m^2^). A-wave and B-wave amplitudes were measured using the Espion analysis software (Diagnosys Technologies, Littleton, MA, USA).

### 2.4. Spectral Domain Optical Coherence Tomography (SD-OCT)

A Spectralis Heidelberg OCT system (Heidelberg Engineering, Heidelberg, Germany) was used for quantitative spectral domain optical coherence tomography (SD-OCT) examination, according to the manufacturer’s instructions and as previously described by our group [31,32]. OCT images (30° field of view) were collected, and retinal thickness was measured at 1500 µm eccentricities from the optic disk, in the four retinal quadrants (nasal, temporal, superior, and inferior). Two parameters were measured, (1) total neuroretina thickness (from nerve fibre layer to RPE) and (2) photoreceptor thickness (from the edge of outer nuclear layer (ONL) to the inner edge of RPE).

### 2.5. Cell Culture and Poly(dA:dT) Transfection

The 661W cell line was generously provided by Dr. Muayyad Al-Ubaidi (Department of Cell Biology, University of Oklahoma Health Sciences Center, Oklahoma City, OK, USA). This cell line is not listed as a commonly misidentified cell line by the International Cell Line Authentication Committee. Experiments utilising 661W photoreceptor cells [33] were seeded at a density of 2.3 × 10^4^ cells/cm^2^ and cultured for 24 h prior to treatment. Fresh 661W medium with additional supplements (as previously described [34]) was then mixed with Poly(dA:dT) (InvivoGen, Toulouse, France) (5 µg/mL: final concentration) and added to 661W cells for up to 8 h. In the WD treatment group, 661W cells were pre-treated with 50 µM of WD for 3 h followed with Poly(dA:dT) treatment for 8 h. Ac-YVAD-cmk (100 µM) (Sigma-Aldrich, Gillingham, UK) was also pre-treated separately in 661W cells for 3 h prior to poly(dA:dT) treatment and then for 8 h in co-treatment. 

### 2.6. YO-PRO™-1 Iodide Assay

YO-PRO™-1 iodide (Thermo Fisher Scientific, Winsford, UK) was used to measure the 661W cell viability. YO-PRO^TM^-1 iodide was diluted (1:2000) in fresh 661W medium (as described above) and was added to different groups of cells in a 96 well flat clear bottom black microplate. YO-PRO™-1 iodide fluorescence was determined by an excitation wavelength of 485 nm and emission wavelength of 520 nm using a POLARstar^®^ Omega microplate reader (BMG Labtech, Ortenberg, Germany). The cell viability was determined by the relative ratio of fluorescence from the treated cells to the control cells.

### 2.7. Reverse Transcriptase Quantitative Polymerase Chain Reaction (RT-qPCR)

The total RNA was extracted using a RNeasy Mini Kit (Qiagen, Hilden, Germany) and the same amount of RNA was transcribed into cDNA using a SuperScript II Reverse Transcriptase Kit (Thermo Fisher Scientific, Winsford, UK), following the manufacturer’s instructions. Validated TaqMan probes from Roche (Roche Holding AG, Basel, Switzerland) were utilised for RT-qPCR (*Aim2* (316827), *Nlrp3* (316820), *Casp1* (300647), *Casp11* (311569), *Il1b* (310471), *Il18* (301115), *Rn18s* (307900), and *Actb* (307903)). RT-qPCR was performed using a Roche LightCycler^®^ 480. The relative gene expression was calculated using the comparative Ct method (2^−ΔCt^) with data normalised to *Actb* or *Rn18s*.

### 2.8. Immunofluorescence

Prior to fixation and microscopy, 661W photoreceptor cells were first seeded at a density of 2.3 × 10^4^ cells/cm^2^ on glass coverslips (VWR international, Radnor, PA, USA) in 24-well plates, and were cultured and treated as above (Cell culture and Poly(dA:dT) transfection). The cells were fixed with 2% paraformaldehyde, permeabilised, and stained with appropriate antibodies (Table 1), as previously described [32]. The cells were mounted using Vectashield medium containing DAPI (Vector Laboratories, Burlingame, CA, USA) and examined using either an Olympus IX51 inverted fluorescent microscope (Olympus, Tokyo, Japan) or Nikon AZ100 multi-zoom microscope (Nikon UK Ltd., Surrey, UK). The images were processed using Fiji software (provided in the public domain, https://imagej.net/Fiji/, accessed on 22 December 2021).

### 2.9. Enzyme-Linked Immunosorbent Assay (ELISA)

Here, 661W cells were seeded at a density of 2.3 × 10^4^ cells/cm^2^ on glass coverslips (VWR international, Radnor, PA, USA) in 24-well plates, and they were cultured and treated as above (cell culture and Poly(dA:dT) transfection). After treatment, the media were removed, centrifuged at 1500 rpm for 5 mins to remove cellular debris, and the supernatant was stored at −20 °C until the experiment. The level of released IL18 in 661W cell supernatants was quantified using a pre-coated ELISA kit (Invitrogen, Paisley, UK) following the manufacturer’s instructions. The absorbance values of each well were measured at 450 nm/570 nm using a POLARstar^®^ Omega microplate reader (BMG Labtech, Ortenberg, Germany).

### 2.10. Statistics

Graphs were created and the statistical analysis was performed using GraphPad Prism (GraphPad Software, San Diego, CA, USA). Unpaired Student’s *t*-test was used to determine the significance between two independent samples. One-way analysis of variance (ANOVA) was used to determine the significance between more than two samples where it was assumed there is normality, sample independence, and variance equality. Tukey’s post-hoc test was used after significance was found between the samples to denote where true differences arose. Data are represented as mean ± standard error of mean (SEM), and a *p* value of less than 0.05 was considered statistically significant.

## 3. Results

### 3.1. WD Attenuates NMU-Induced Photoreceptor Degeneration

**Figure 1:**
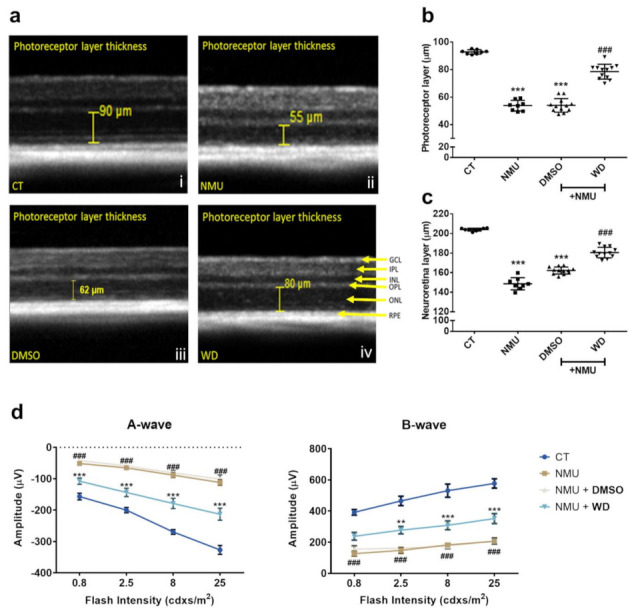
(**a**) Representative SD-OCT images derived from Heidelberg Spectralis software, displaying manual photoreceptor layer measurement (yellow measurement bar). (**b**) Quantitative analysis of the average photoreceptor layer thickness and (**c**) average neuroretina thickness was taken from four measurements (nasal, temporal, superior, and inferior) at 1500 µm eccentricities from the optic disk, per eye. CT: *n* = 8, NMU: *n* = 8, NMU + DMSO: *n* = 12, NMU + WD: *n* = 12; *n* = number of individual mouse eyes. One-way ANOVA with Tukey’s post hoc test. *** *p* < 0.001 comparison to CT. ### *p* < 0.001 comparison between NMU + WD and NMU + DMSO. (**d**) Analysis of A- and B-wave amplitude. The retinas from each experimental group were stimulated with increasing light intensities (0.8, 2.5, 8, and 25 cdxs/m^2^). CT: *n* = 6, NMU: *n* = 10, NMU + DMSO: *n* = 8, NMU + WD: *n* = 12; *n* = number of individual mouse eyes. Two-way ANOVA with Tukey’s post hoc test ** *p* < 0.01, *** *p* < 0.001 comparison between NMU + WD and NMU + DMSO. ### *p* < 0.001 comparison between NMU and CT. Error bars: mean ± SEM.

The structural integrity of the retinas from all experimental groups were examined using SD-OCT. At 7 days post NMU injection (40 mg/kg), there was a significantly reduced neuroretina thickness (Figure 1a) with the cell-loss predominately occurring in the photoreceptor layer (Figure 1b,c) (*p* < 0.001). The thickness of the entire neuroretina and the photoreceptor layer was significantly improved in the WD treated mice compared to that in the NMU or NMU + DMSO vehicle-treated mice (Figure 1a–c). There was no significant difference in retinal thickness or photoreceptor thickness between NMU alone and the NMU + DMSO group (Figure 1a–c). The visual function was evaluated by ERG. Seven days post NMU treatment, both A- and B-wave amplitudes were significantly reduced in the NMU-treated mice compared to the naïve control mice, at all light intensities (Figure 1d, *p* < 0.001). WD treatment significantly improved A-wave amplitude at all light intensities and B-wave at the light intensity of 2.5 cdxs/m^2^ and above when compared to the NMU + DMSO group (Figure 1d, *p* < 0.001). There was no difference between NMU and NMU + DMSO groups in both the A- and B-wave responses. Together, our results suggest that WD strongly protected photoreceptors from NMU-induced neurodegeneration.

### 3.2. Effect of WD on NMU-Induced Retinal Inflammasome Activation

**Figure 2:**
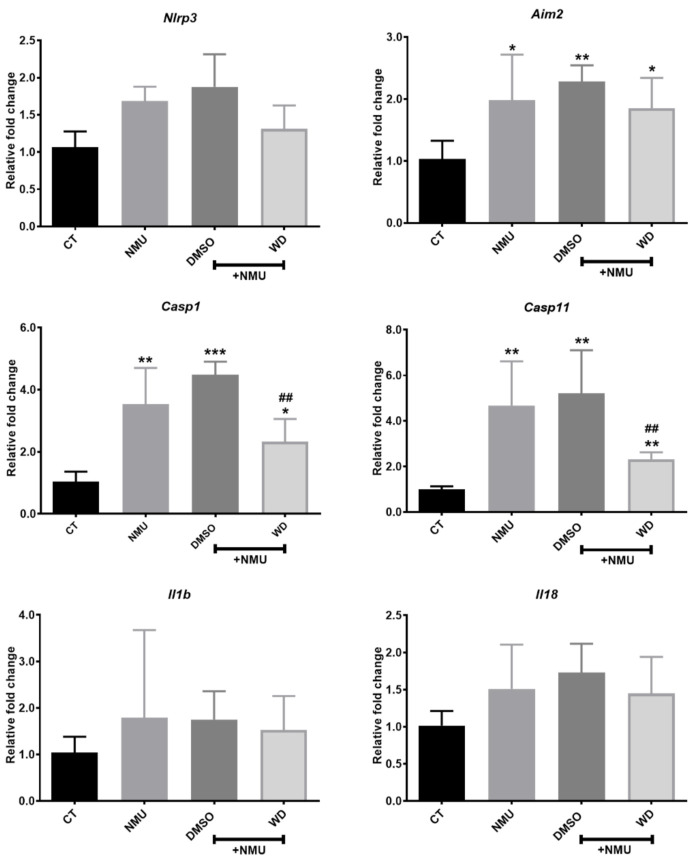
Three-month old mice were injected with NMU in combination with either WD or DMSO. After 7 days, the retinas were enucleated from mice and processed for mRNA expression of the inflammasome components. Relative mRNA expression of inflammasome components compared to the reference gene Rn18s. CT: *n* = 4, NMU: *n* = 6, NMU + DMSO: *n* = 6, NMU + WD: *n* = 6; *n*: the number of mouse eyes per group. One-way ANOVA with Tukey’s post-hoc test * *p* < 0.05, ** *p* < 0.01, and *** *p* < 0.001: comparison to CT. ## *p* < 0.01: comparison between NMU + WD and NMU + DMSO. One-way ANOVA with Tukey’s post-hoc test. Error bars shown as mean ± SEM.

To understand the mechanism of WD-mediated photoreceptor protection, we investigated inflammasome activation in NMU-induced retinal neurodegeneration. RT-qPCR showed that *Aim2*, *Casp11*, and *Casp1* were significantly upregulated in the NMU treated mouse retinas compared to those in the retinas from naïve control mice (Figure 2; *p* < 0.05). There was no significant change in the expression of *Nlrp3*, *Il18*, and *Il1b* mRNA in the NMU-treated mice (Figure 2). WD treatment significantly reduced the NMU-induced upregulation of *Casp11* and *Casp1* mRNA (Figure 2; *p* < 0.01) but did not affect the NMU-induced upregulation of *Aim2* (Figure 2). The vehicle DMSO did not affect any of the NMU-induced upregulation of the inflammasome component (Figure 2). Western blotting was additionally carried out on several inflammasome components (data no shown); however, there was no statistical significance found between the individual proteins in the various treatment groups. Our results suggest that NMU-induced retinal inflammasome activation can be partly attenuated by WD at the mRNA level.

### 3.3. Poly(dA:dT)-Induced 661W Photoreceptor Cell Death Is Associated with the Aim2/Casp11/Il18 Signalling Cascade, and Is Casp1 Independent

**Figure 3:**
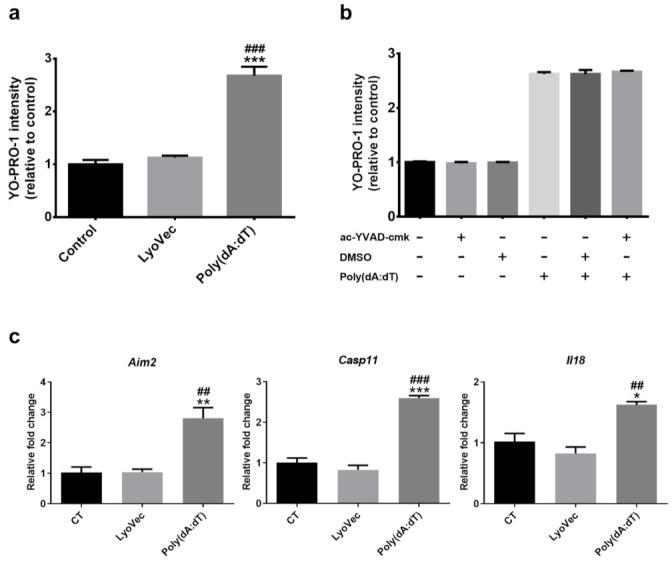
(**a**) 661W photoreceptor cells were incubated with Poly(dA:dT) or LyoVec (Poly(dA:dT) loading vector control), and (**b**) Poly(dA:dT) +/− ac-YVAD-cmk (100 µM) (Casp1 inhibitor) or DMSO (0.01%) for 8 h. The cell viability of 661W photoreceptor cells was determined by YO-PRO-1 uptake after 8 h of treatment. (**c**) Relative mRNA expression of inflammasome components in 661W photoreceptor cells after 8 h Poly(dA:dT) treatment, compared to the reference gene *Actb*. *n* = 3 per group; *n* represents the average of three experimental repeats. One-way ANOVA with Tukey’s post-hoc test. * *p* < 0.05, ** *p* < 0.01, and *** *p* < 0.001: comparison to the control. ## *p* < 0.01 and ### *p* < 0.001: comparison to LyoVec. Error bars shown as mean ± SEM.

To understand the mechanism of NMU-induced- inflammasome and photoreceptor death, we used the synthetic double-stranded DNA (dsDNA), Poly(dA:dT), to mimic cytosolic DNA released from the damaged mitochondria. Poly(dA:dT) transfection resulted in a significantly higher YO-PRO-1 uptake compared to the controls (Figure 3a, *p* < 0.001), indicative of reduced cell viability. Surprisingly, a selective Casp1 inhibitor, ac-YVAD-cmk, failed to protect 661W cells from Poly(dA:dT) induced death (Figure 3b). The RT-qPCR analysis showed that the expression of *Aim2*, *Casp11*, and *Il18* was significantly upregulated following Poly(dA:dT) treatment (Figure 3c). The expression of *Casp1* and *Il1b* was merely detectable (Ct value > 35) in 661W cells with/without Poly(dA:dT) treatment (data not shown). Our results suggest that Poly(dA:dT) might induce photoreceptor death through activating the Aim2/Casp11/Il18 inflammasome pathway.

### 3.4. WD Attenuated Poly(dA:dT)-Induced Photoreceptor Cell Death through Down Regulation of the Aim2/Casp11/Il18 Pathway

**Figure 4:**
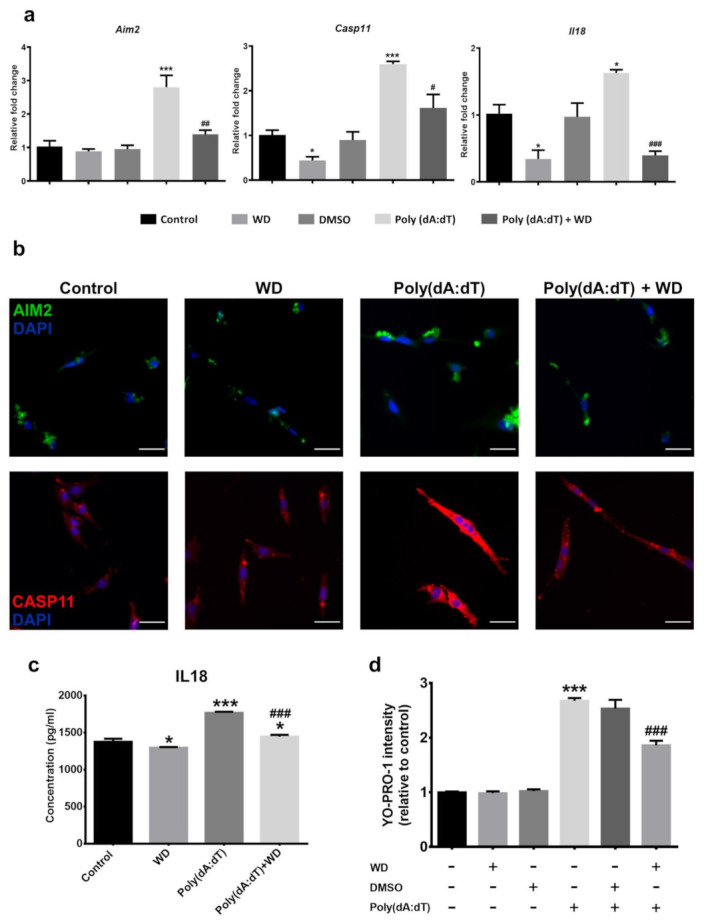
661W photoreceptor cells were incubated with Poly(dA:dT) (5 µg/ml) ± WD (50 µM) or DMSO (0.01%) for 8 h prior to analysis. (**a**) Relative mRNA expression of inflammasome components in 661W photoreceptor cells, compared to the reference gene Actb. *n* = 3 per group; *n* represents the average of three experimental repeats. (**b**) Representative immunocytochemistry images of AIM2 (green) and CASP11 (red) taken from treated 661W photoreceptor cells, counterstained with DAPI (blue) to visualise the nucleus. Scale bar: 50 µm. (**c**) The relative protein expression and release of inflammatory cytokine IL18 was quantified through ELISA. The results were calculated by interpolating the absorbance values against a standard curve of known IL18 concentrations. *n* = 3 per group; *n* represents the average of three experimental repeats. (**d**) Cell viability of 661W photoreceptor cells was determined by YO-PRO-1 uptake after 8 h of treatment; *n* = 6 per group. One-way ANOVA with Tukey’s post-hoc test. * *p* < 0.05 and *** *p* < 0.001: relative to the control. # *p* < 0.05, ## *p* < 0.01, ### *p* < 0.001: relative to Poly(dA:dT). Error bars shown as mean ± SEM.

To understand if WD can protect photoreceptors from Poly(dA:dT)-induced death, we pre-incubated 661W cells with 50 µM of WD for 3 h prior to Poly(dA:dT) treatment. The RT-qPCR analysis showed that WD treatment significantly attenuated the Poly(dA:dT)-induced upregulation of *Aim2*, *Casp11*, and *Il18* mRNA (Figure 4a). Immunofluorescent staining showed low levels of CASP11 (green) and AIM2 (red) expression in 661W cells under normal culture conditions (Figure 4b). Their expression was markedly enhanced by Poly(dA:dT), but not WD treatment (Figure 4b). Poly(dA:dT)-induced the upregulation of AIM2 and CASP11 was attenuated by WD pre-incubation (Figure 4b). IL18 was constitutively produced by 661W cells under normal culture conditions (Figure 4c). Poly(dA:dT) treatment significantly elevated IL18 production (*p* < 0.001), and this was attenuated by WD (Figure 4c; *p* < 0.001). WD alone sightly inhibited IL18 production (*p* < 0.05). WD on its own did not affect 661W cell viability (Figure 4d), however it significantly reduced Poly(dA:dT)-induced 661W cell death.

## 4. Discussion

NMU is a DNA alkylating agent that damages the molecular integrity of DNA, leading to the development of various cancers and retinal degeneration [35,36,37]. NMU-induced retinal neurodegeneration has been used as a model of oxidative stress and inflammation-related retinopathies [38,39], although the underlying mechanism of cell death post-DNA damage remains poorly defined. In this study, we show that *Aim2/Casp11/Il18* inflammasome activation plays a key role in NMU-induced photoreceptor death. We further show that WD protected NMU-induced retinal neurodegeneration, which was achieved, at least partially, through the suppression of NMU-induced inflammasome activation.

Photoreceptor cells contain a high abundance of mitochondria, which are vulnerable to oxidative stress-induced mitochondrial dysfunction [35]. The AIM2 inflammasome primarily senses DNA, which induces its activation [40,41,42]. NMU treatment may lead to cytosolic release of mtDNA and nuclear DNA, which can induce AIM2-related inflammasome activation, including downstream CASP1 and CASP11 activation, and subsequently the maturation of IL-1 family cytokines (IL1B and IL18). This hypothesis was further reinforced by our parallel in vitro study, whereby the transfection of synthetic dsDNA Poly(dA:dT) induced AIM2 inflammasome activation and concomitantly increased the expression of IL18 in 661W photoreceptor cells. Wooff et al. found that the mRNA expression of *Nlrp3, Casp1,* and *IL1b* in 661W cells were relatively low when compared to other retinal cells such as MIO-M1 Müller cells [43]. We also observed a similar pattern in our study (data not shown). This indicates that IL1B might not be the predominant cytokine inducing photoreceptor death. The failed protection of the selective CASP1 inhibitor from Poly(dA:dT)-induced 661W cell death might be partially due to the low level of CASP1 and IL1B. It is notable that abnormal levels of IL18 can cause dry-AMD type retinal degeneration; however, this was observed in an in vitro model with retinal pigment epithelium (RPE) [44].

Another important observation of our study is that WD can protect the retina from NMU-induced neurodegeneration, partially through the suppression of AIM2 inflammasome activation. WD is chemically obtained from *Eclipta alba*, a common plant-derived traditional medicine [45]. WD has been shown to inhibit CASP11, a major regulator of proinflammatory cytokine IL1B maturation [27,28]. This was confirmed in our study in NMU-treated mouse retinas and Poly(dA:dT) treated 661W cells. In addition, WD can also suppress inflammation through the inhibition of IKKγ, a kinase that is crucial for the activation of NF-κB, as well as IKK α and IKK β [27]. Furthermore, WD also has anti-oxidative effects [25,46], which may contribute to its protective effect on NMU-induced photoreceptor degeneration. It has been previously reported that the non-canonical activation of CASP11 may regulate CASP1 activation through the NLRP3/ASC canonical pathway [47]; therefore, WD may modulate CASP1 indirectly via CASP11 inhibition. CASP1 and CASP11 are well-established IL-1 converting enzymes [41,48,49]. WD inhibition of CASP11 in the retina of NMU-induced neurodegeneration importantly showed a reduction in the expression of IL18. IL18 has been implicated in several ocular diseases, including AMD [50,51], retinopathy of prematurity [52], and glaucoma [53]. Interestingly, IL18 has shown conflicting results within these models (particularly AMD), where it has demonstrated pro- and anti-inflammatory properties in the retinal pigmented epithelium [54]. Our study focused on the retinal expression of IL18, where we show that increased levels contribute to neurodegeneration. Targeting upstream inflammasome signaling, from IL18, may prove to be a viable therapeutic option in the retina; however, there should be careful consideration with respect to the dual properties of IL18. In this study, NMU-induced photoreceptor degeneration was not completely attenuated by WD administration via the AIM2/CASP11 axis, indicating that multiple mechanisms contribute to the induction of photoreceptor cell death. Recent studies have also suggested that NMU induces photoreceptor death via the modulation of SIRT1, ERS, and apoptosis [55].

During our research, the beneficial effects of WD as an anti-inflammatory drug in various models, where NLRP3 inflammasome is activated, have been published [56,57,58]. However, we believe our study provides the first indication that WD plays a protective and anti-inflammatory role in the retina, specifically through the AIM2/CASP11 inflammasome cascade. Additionally, the application of WD in inflammation-mediated retinal photoreceptor degeneration may create a valuable therapeutic avenue.

## 5. Conclusions

In this murine study, we show that AIM2 inflammasome activation is critically involved in NMU-induced retinal photoreceptor degeneration and that WD treatment offers cytoprotection, which is, in part, achieved through the suppression of AIM2 inflammasome activation. WD shows promising in vitro and in vivo efficacy as a viable therapeutic in the treatment of retinal neurodegenerative diseases.

## Figures and Tables

**Table 1:** List of primary and secondary antibodies utilised for immunofluorescence.

Antibody	Concentration	Company	Catalogue ID	Species
AIM2	1:50	Abcam	AB93015	Rb
CASP11	1:50	Thermofisher Scientific	PA5-20108	Rb
Donkey anti-Rabbit Alexa Fluor 488	1:300	Jackson ImmunoResearch	711-545-152	
Donkey anti-Rabbit Alexa Fluor 594	1:300	Jackson ImmunoResearch	711-585-152	

## Data Availability

The datasets used during the current study are available from the corresponding author upon reasonable request.
